# The Incidence of Rectal Neuroendocrine Tumors Is Increasing in Younger Adults in the US, 2001–2020

**DOI:** 10.3390/cancers15215286

**Published:** 2023-11-04

**Authors:** Yazan Abboud, Navya Pendyala, Alexander Le, Anmol Mittal, Saqr Alsakarneh, Fouad Jaber, Kaveh Hajifathalian

**Affiliations:** 1 Department of Internal Medicine, Rutgers New Jersey Medical School, Newark, NJ 07103, USA; np726@njms.rutgers.edu (N.P.); al1424@njms.rutgers.edu (A.L.); am1777@njms.rutgers.edu (A.M.); 2 Department of Internal Medicine, University of Missouri-Kansas City, Kansas City, MO 64108, USA; s.alsakarneh@umkc.edu (S.A.); fouad.jaber@umkc.edu (F.J.); 3 Division of Gastroenterology and Hepatology, Rutgers New Jersey Medical School, Newark, NJ 07103, USA; kh852@njms.rutgers.edu

**Keywords:** rectal neuroendocrine tumors, incidence, epidemiology, rectal cancer, health disparity, neuroendocrine tumors

## Abstract

**Simple Summary:**

Prior data showed an increasing incidence of rectal neuroendocrine tumors (RNET) in the US. There are limited comprehensive recent data on RNET incidence and time-trends among demographic-specific populations. The aim of this study was to evaluate recent age-specific RNET incidence rates and time-trends in demographic- and tumor-specific populations, using the United States Cancer Statistics (USCS) data covering ~98% of the US population between 2001 and 2020. Our nationwide analysis including 59,846 patients diagnosed with RNET shows a significantly increasing incidence of RNET in younger adults. An age-specific comparative analysis showed a significantly greater increase in younger adults compared to older adults. A sex-specific analysis showed that the increase was mostly driven by younger women and by tumors diagnosed at an early stage. The age-specific difference in RNET incidence was noted in various races. A sensitivity analysis of microscopically confirmed RNET cases showed similar results to the overall analysis. Our study provides comprehensive epidemiological data aiming to guide further investigations on this emerging topic.

**Abstract:**

Prior non-comparative data showed increasing incidence of rectal neuroendocrine tumors (RNET) in the US. We aimed to evaluate age-specific RNET incidence rates and time-trends in demographic- and tumor-specific populations. The RNET age-adjusted incidence rates were calculated from the United States Cancer Statistics (USCS) database between 2001 and 2020. The population was stratified by age into older (≥55 years) and younger adults (<55 years), as well as by sex and race. The tumors were categorized by their stage at diagnosis into early and late. The annual percentage change (APC) and average APC (AAPC) were estimated using joinpoint regression and Monte Carlo permutation analysis. Pairwise comparison assessed for parallelism and coincidence. There were 59,846 patients diagnosed with RNET between 2001 and 2020 (50.3% women). Overall, the RNET incidence rates during this period were increasing in younger but not older adults (AAPC = 3.12 vs. −1.10; AAPC difference = 4.22, *p* < 0.001), with non-identical non-parallel data (*p*-values < 0.001). While similar results were seen in men, a greater age-specific difference was noted in women (AAPC = 3.31 vs. −1.10; AAPC difference = 4.41, *p* = 0.003). The difference between younger and older adults was seen in non-Hispanic White (AAPC-difference = 4.89; *p* < 0.001) and non-Hispanic Black (AAPC-difference = 3.33; *p* = 0.03) patients, and, in most years, among Hispanic and Non-Hispanic Asian/Pacific Islander patients, and it was mostly driven by early-stage tumors (AAPC-difference = 3.93; *p* < 0.001). The nationwide data show a significantly increasing RNET incidence in younger adults, most notably in younger women and in early-stage tumors, seen in various races. Future studies should evaluate RNET risk factors and outcomes in demographic-specific populations.

## 1. Introduction

Neuroendocrine tumors are a group of neoplasms that can exhibit dichotomous functions mimicking both the characteristics of nerve cells and the hormone-secreting capabilities of endocrine cells [1]. The incidence of neuroendocrine tumors was shown to be increasing in a previous analysis of the Surveillance Epidemiology and End Results (SEER) database in multiple body organs, including several parts of the gastrointestinal tract [2]. Prior theories suggested that the increase in incidence could be the result of the improvement in detection modalities, including imaging and endoscopic procedures. The small intestine has been considered the most common site for neuroendocrine tumors in the past; however, after the implementation of the new colonoscopy screening recommendations by the American College of Gastroenterology, rectal neuroendocrine tumors (RNET) are at least as prevalent as small intestine carcinoid tumors [3]. RNET are mostly asymptomatic and found incidentally [4], with an incidence rate of 0.17% during screening colonoscopies [5]. A previous nationwide analysis evaluating 4918 RNET cases showed an increasing RNET incidence between 1992 and 2015. However, the study was limited in such that it only covered 13.4% of the US population, which may limit the generalizability of the findings. Furthermore, the study did not provide comparative data of age-specific trends, nor evaluated the rates or trends by race or tumor characteristics [6]. Despite the growing literature investigating RNET, there are limited data on recent RNET age- and sex-specific incidence rates. Therefore, the aim of this study was to evaluate recent RNET incidence rates and time-trends among population-specific demographics and tumor-specific characteristics using a nationwide comprehensive database, the United States Cancer Statistics (USCS) database [7]. We aimed to evaluate the following:

- The RNET incidence rates and time-trends in age- and sex-specific populations;
- The impact of race on RNET incidence rates and time-trends in different age groups;
- The impact of a tumor’s stage at diagnosis on the RNET incidence rates and time-trends in different age groups.

The findings of this study were presented in part as a lecturer presentation at the Digestive Diseases Week (DDW) 2023 conference at the “AGA Colorectal Cancer Screening and Surveillance: High-Risk Populations, Including Hereditary Syndromes and Inflammatory Bowel Disease” session on the 6th of May 2023 (10:45 A.M. to 11:00 A.M.), in Chicago, IL. Other findings of this study were accepted at the American College of Gastroenterology Annual Scientific Meeting & Postgraduate Course and were presented on the 22nd of October 2023 in Vancouver, Canada.

## 2. Materials and Methods

This is a nationwide population-based time-trend analysis of RNET incidence rates in the US between 2001 and 2020, using the USCS database. The data were de-identified and publicly available, and, therefore, based on the National Human Research Protections Advisory Committee Policy, the data were exempted from review by the institutional review board.

### 2.1. Data Collection

The RNET incidence rates between the 1st of January 2001 and the 31st of December 2020 were collected from the USCS database, a comprehensive source of cancer incidence statistics in the US, which nearly covers 98% of the US population [7]. The USCS database has data from the Centers for Disease Control and Prevention (CDC)’s National Program of Cancer Registries (NPCR) and from the National Cancer Institute (NCI)’s Surveillance, Epidemiology, and End Results (SEER) program. It covers all 50 states, the District of Columbia, and Puerto Rico, providing data on nearly 33 million cancer cases [7]. All collected data by the US cancer registries get implemented into automated software programs to maintain high-quality standardization and coding as per the North American Association of Central Cancer Registries’ data standards [8].

### 2.2. Definitions

The RNET incidence rate was defined as the number of patients diagnosed with RNET per 100,000 population each year. The annual percentage change (APC) was defined as the percentage change in the RNET incidence rates between two years. The average APC (AAPC) was defined as the average percentage change in the RNET incidence rates between 2001 and 2020. Increasing and decreasing trends were defined as the statistically significant positive and negative values of the AAPC, respectively, while the non-statistically significant changes in the AAPC were identified as stable trends. The tumors’ location was specified as being in the “Rectum and Rectosigmoid Junction”, with a malignant behavior. The *International Classification of Diseases for Oncology*, Third Edition, Site Record ICD-O-3/WHO 2008, was used to identify RNET codes as follows: 8240, 8241, 8245, 8246, and 8249 [9]. The population was divided using a cutoff age of 55 years into the following two pre-specified age groups, as defined in prior studies [10,11]: older adults, i.e., patients aged 55 years or older, and younger adults, i.e., patients aged 15–54 years (<55 years). The population was also categorized by sex and by race into the following groups, as defined in the database: Hispanic (H), Non-Hispanic Black (NHB), Non-Hispanic White (NHW), Non-Hispanic Asian/Pacific Islander (NHAPI), and Non-Hispanic American Indian/Alaska Native (NHAIAN). The tumors’ stage at diagnosis was defined as early stage, including in situ and localized tumors, and late stage, including tumors with regional or distant site/nodes’ involvements.

### 2.3. Statistical Analysis

The RNET incidence rates were calculated and adjusted for age based on the 2000 standard US population using the SEER*Stat software (v.8.4.1.2, National Cancer Institute “NCI”). The Joinpoint Regression Software (v.4.9.0.1, NCI) was used to analyze the time-trends, which were estimated as the APC and the AAPC. This software uses Monte Carlo permutation analysis to identify the simplest trend that reflects the change in rates over time [12,13]. Using tests of parallelism and coincidence, a pairwise comparison was conducted between the age-specific trends, and the absolute AAPC difference was evaluated [14]. Further analysis was conducted in sex- and race-specific populations and, also, after categorizing the tumors by their stage at diagnosis. Lastly, a sensitivity analysis was conducted using microscopically confirmed cases only. A two-sided *p*-value cutoff at 0.05 was utilized for statistical significance. 

## 3. Results

### 3.1. RNET Incidence Rates and Time-Trends in Age- and Sex-Specific Populations

**Figure 1:**
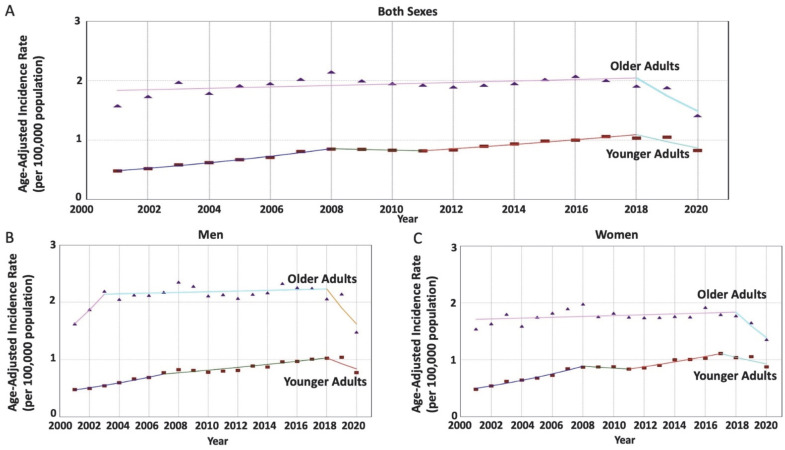
Age-specific time-trends of incidence rates per 100,000 population for rectal neuroendocrine tumors (RNET) among men and women. (**A**) The average annual percentage change (AAPC) is increasing in younger adults at a greater rate compared to the stable trend in older adults, with a significant difference (3.12 vs. −1.10, *p* < 0.001). (**B**) The average annual percentage change (AAPC) is increasing in younger men at a greater rate compared to the stable trend in older men, with a significant difference (3.08 vs. −0.02, *p* = 0.01). (**C**) The average annual percentage change (AAPC) is increasing in younger women at a greater rate compared to the stable trend in older men, with a significant difference (3.31 vs. −1.10, *p* = 0.003).

From 2001 to 2020, there were 59,846 cases of RNET diagnosed in the US. Notably, RNET incidence rates have been significantly increasing in younger adults in contrast to the stable trend that has been maintained in older adults (AAPC = 3.12 vs. −1.10; AAPC difference = 4.22, *p* < 0.001). The age-specific trends during this period were not uniform (*p* < 0.001), nor parallel (*p* < 0.001), suggesting that the RNET incidence rates in younger adults are distinct and rising at a greater rate compared to older adults (Table 1). This trend was replicated in men (29,772 patients), with a greater increase in RNET incidence rates among younger adults compared to older adults (AAPC = 3.08 vs. −0.02; AAPC difference = 3.10, *p* = 0.01). While similar results were seen in women (30,074 patients), a greater AAPC difference between younger and older adults was noted (AAPC = 3.31 vs. −1.10; AAPC difference = 4.41, *p* = 0.003), with non-parallel (*p* < 0.001) non-identical (*p* < 0.001) data. This underscores women as the population exhibiting the greatest disparity between the RNET incidence trends between age-specific groups (Figure 1).

### 3.2. RNET Incidence Rates and Time-Trends in Age- and Race-Specific Populations

**Figure 2:**
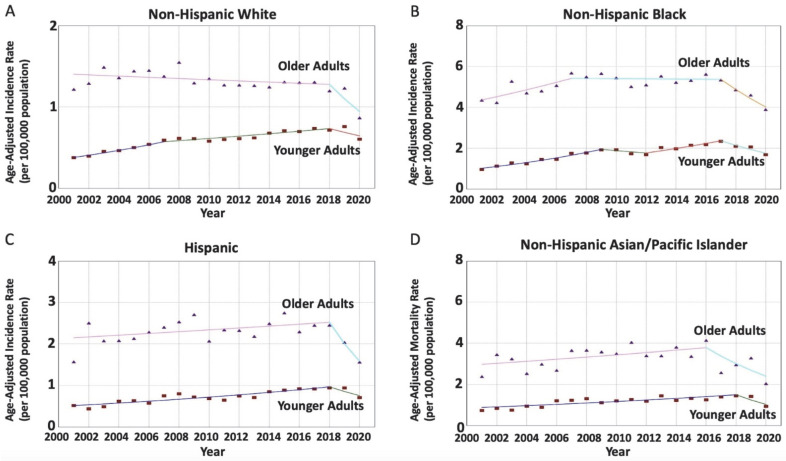
Age-specific time-trends of incidence rates per 100,000 population for rectal neuroendocrine tumors (RNET) among different race groups. (**A**) The average annual percentage change (AAPC) is increasing in younger NHW patients while decreasing in older NHW patients, with a significant difference (2.83 vs. −2.06; *p* < 0.001) and non-parallel (*p* < 0.001) non-identical (*p* < 0.001) trends. (**B**) The average annual percentage change (AAPC) is increasing in younger NHB patients while remaining stable in older NHB patients, with a significant difference (2.90 vs. −0.43; *p* = 0.03) and non-parallel (*p* < 0.001) non-identical (*p* < 0.001) trends. (**C**) The average annual percentage change (AAPC) is stable in younger H patients and older H patients (2.03 vs. −1.59; *p* = 0.005), with non-parallel (*p* < 0.001) non-identical (*p* < 0.001) trends. (**D**) The average annual percentage change (AAPC) is increasing in younger NHAPI patients while remaining stable in older NHAPI patients, with a significant difference (0.81 vs. −1.14; *p* = 0.36) and parallel (*p* = 0.15) non-identical (*p* < 0.001) trends.

In NHW patients (29,720 patients), the RNET incidence rates were significantly increasing in younger adults and decreasing in older adults (AAPC = 2.83 vs. −2.06; AAPC difference = 4.89, *p* < 0.001). In NHB patients (16,025 patients), the incidence rates were also increasing in younger adults but not in older adults (AAPC = 2.90 vs. −0.43; AAPC difference = 3.33, *p* = 0.03). In H patients (6903 patients), while the RNET incidence rates were increasing only in younger adults between 2001 and 2018 (APC = 3.77 vs. 0.95), the rates were stable in both age groups between 2018 and 2020. The AAPCs were non-identical (*p* < 0.001) and non-parallel (*p* < 0.001), with an AAPC difference of 3.62 (*p* = 0.05), suggesting that the rates in younger adults were different than older adults and that the absolute difference was trending to be statistically significant. In NHAPI patients (4577 patients), while the rates were increasing in younger adults between 2001 and 2018 (AAPC = 3.16) and stabilized afterward, older adults experienced stable trends during the study period, with parallel data (*p* = 0.15) and a non-significant difference (*p* = 0.36). Lastly, in NHAIAN patients, there were 425 patients who were diagnosed with RNET, but the number of yearly cases was too small to estimate a trend (Table 2 and Figure 2).

### 3.3. RNET Incidence Rates and Time-Trends in Age-Specific Populations Characterized by Tumors’ Stage at Diagnosis

**Figure 3:**
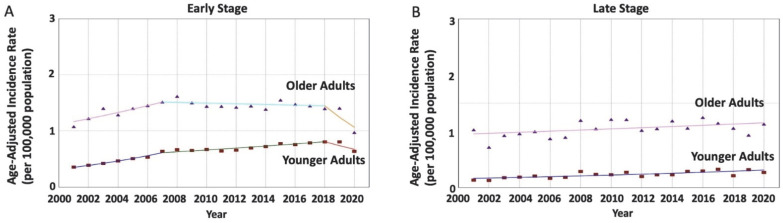
Age-specific time-trends of incidence rates per 100,000 population for rectal neuroendocrine tumors (RNET) characterized by tumors’ stage at diagnosis. (**A**) For tumors diagnosed at an early stage, the average annual percentage change (AAPC) is increasing in younger adults but not in older adults, with a significant difference (3.47 vs. −0.46; *p* < 0.001) and non-parallel (*p* < 0.001) non-identical (*p* < 0.001) trends. (**B**) For tumors diagnosed at a late stage, the average annual percentage change (AAPC) is increasing in younger adults at a significantly greater rate compared to older adults (3.43 vs. 0.99; *p* = 0.004) with non-parallel (*p* < 0.001) non-identical (*p* < 0.001) trends.

In tumors diagnosed at an early stage (45,146 patients), the RNET incidence rates were increasing in younger adults but not in older adults (AAPC = 3.47 vs. −0.46; AAPC difference= 3.93, *p* < 0.001). The age-specific trends were non-identical (*p* < 0.001) and non-parallel (*p* < 0.001), suggesting that the rates in younger adults were increasing at a greater rate compared to older adults. In tumors diagnosed at a late stage (4012 patients), the RNET incidence rates were increasing in younger adults at a greater rate compared to older adults (AAPC = 3.43 vs. 0.99; AAPC difference = 2.44, *p* = 0.004), with non-identical (*p* < 0.001) non-parallel data (*p* < 0.001) as well (Table 2 and Figure 3).

### 3.4. Sensitivity Analysis

**Figure 4:**
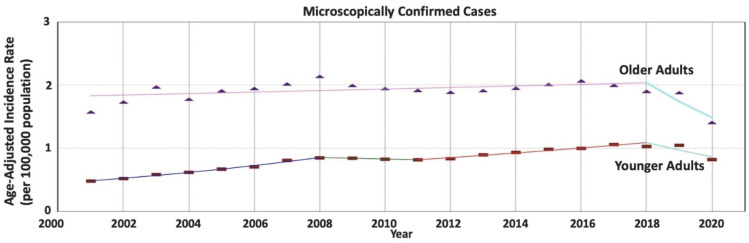
Age-specific time-trends of incidence rates per 100,000 population for microscopically confirmed rectal neuroendocrine tumors (RNET). The average annual percentage change (AAPC) is increasing in younger adults at a greater rate compared to the stable trend in older adults, with a significant difference (3.10 vs. −1.10, *p* < 0.001).

Our sensitivity analysis of microscopically confirmed RNET cases shows similar results to the overall analysis, showing a greater increase in the RNET incidence rates in younger adults compared to older adults (AAPC = 3.10 vs. −1.10; AAPC difference = 4.20; *p* < 0.001) (Table 3 and Figure 4).

## 4. Discussion

Our nationwide study evaluating nearly all patients diagnosed with RNET in the US between 2001 and 2020 showed a significant increase in the RNET incidence rates in younger adults aged <55 years when compared to older adults aged ≥55 years. Our analysis by sex and by race showed that the largest disparity between younger and older adults was arising from women’s rates and was seen in NHW and NHB patients, as well as in H and NHAPI patients in most years. When characterizing the tumors by stage at diagnosis, the greatest difference between the age-specific trends was seen in the tumors diagnosed at an early stage. 

The rising incidence of NETs has been widely established in the literature, with a 6-fold increase in the US over the last three decades [15]. However, there are limited data evaluating the increased incidence of rectal NETs in recent years while also identifying differences in the incidence rates across different sexes, age groups, and racial populations. A previous non-comparative SEER-based analysis found that there has been a rising incidence of RNETs from 1975 to 2015 [6] While the previous study shows similar findings to our current study, there are several differences. Our study offers a significantly larger sample size (59,846 patients vs. 4918 patients), provides an age-specific comparative analysis, evaluates updated data between 2001 and 2020, and provides a sensitivity analysis of microscopically confirmed cases. We also categorized the age-specific analysis by sex and showed that the largest difference between younger and older adults was arising from women. 

This increase in RNET incidence may in part be ascribed to an increase in the breadth of knowledge known about GI NETs, as demonstrated by new classifications of the tumors defined by the WHO as recently as 2019 [16]. Although there has been little research on the role of more developed imaging techniques, such as PET radiography using gallium-based tracers which act as somatostatin analogs for RNET diagnosis specifically, these techniques have been proven to be effective and a contributing factor to the rising incidence of numerous GI NETs, including RNET [17,18]. This could also be a possible reason driving the increase in RNET in recent years. Another possible explanation for the increased RNET incidence has been attributed to growing awareness of these tumors and advancements in endoscopy utilization for cancer screening, and, thus, diagnosing a wider population range [19]. Procedural advancements in recent years have allowed for increased detection of smaller lesions or precursor lesions, potentially explaining the increased AAPC in younger adults when compared to older adults [6]. It was hypothesized that this effect would eventually plateau, as it can be seen from our data showing a stable trend of RNET incidence in older adults [20]. Having said that, prior data showed that women are less likely to obtain screening colonoscopies compared to men [21,22], which does not align with the prior theories of increased detection of RNET, given that the greater increase was seen in younger women. Considering the differences in AAPC between men and women, it is crucial to further investigate the reasoning behind this, while also identifying the differences in risk factors which may contribute to missed diagnoses.

There is a growing body of literature showing racial disparities in RNET incidence rates. Previous data showed that the most common location of NET in African American, American Indian/Alaskan Native, and Asian/Pacific Islander patients was the rectum [23]. Prior data also showed that the increasing incidence of RNET was more prominent in the African American population [9]. Our findings show that the RNET incidence rates were highest in all racial minorities (NHB, H, and NHAPI patients) compared to the NHW population, consistent with the literature [3]. However, our study provides an age-specific analysis of different race groups and shows that the greatest AAPC in different age- and race-specific cohorts was found in younger NHB patients (AAPC = 2.90). We also show that younger Hispanic patients and younger NHAPI patients experienced a significant increase in RNET incidence rates between 2001 and 2018, with an AAPC of 3.77 and 3.16, respectively. Our findings suggest an overall greater increase in younger adults compared to older adults in NHW, NHB, and H patients. Our study also shows that the RNET incidence rates in older adults were decreasing in NHW patients and remained stable in all the other racial groups. Many may attribute the increase in RNET incidence in the various population groups to the increase in colonoscopy screening, but this seems to only hold true for age and population as a whole. It has been found that ethnic minorities are less likely to obtain screening colonoscopies [24], suggesting that there must be another reason for the disparities within the various demographic characteristics which must be further investigated [3].

With regard to the stage at diagnosis, prior nationwide data from Canada suggested that the increasing incidence of RNET was driven by tumors diagnosed at an early stage [25]. Our study adds to the existing literature by providing comprehensive US data on RNET incidence categorized by the stage at diagnosis in different age groups. We demonstrate an increasing incidence of early- and late-stage RNET, with the most significant increase in tumors diagnosed at an early stage in younger adults and significant differences between age-specific groups. This could be the result of the increased detection of those tumors due to improvements in diagnostic modalities such as computed tomography and endoscopic procedures. It has also been postulated that these findings may partly reflect changes in the diagnostic criteria or changes in tumor biology [6]. However, the data on these theories are very limited, and, ultimately, a true increase in the tumors’ incidence cannot be ruled out.

Some of the strengths of our study include the large sample size in the USCS database (59,846 patients; ~98% of the US population) and the use of joinpoint regression to conduct time-trend analysis, which is recommended in large databases [26]. Furthermore, we demonstrated an age-specific comparative analysis of RNET incidence rates between older and younger adults. We also provided an analysis categorized by patients’ race and tumors’ stage at diagnosis, with the goal of better understanding the epidemiology of RNET in different populations. In addition, we performed a sensitivity analysis of microscopically confirmed cases and found similar results to the overall analysis. With that in mind, our study suffers from several limitations. First, our study is observational in nature and hypothesis-generating, which limited us from identifying any risk factors for the revealed findings. Second, the coding reliability and possible loss of records are some inherent limitations of the SEER database that can be implied for the NPCR database, given the similar methodology utilized for collecting data between the two databases [27]. Therefore, those limitations can be generalized to the USCS database. However, the USCS database is the official source of federal cancer statistics in the US. It is a high-quality database and undergoes rigorous quality checks and reviews before publication to minimize any human errors [7]. Similar to much of the new literature establishing an increasing incidence of gastrointestinal cancers, such as colorectal cancer [28], gastric cancer [29], and pancreatic cancer [10,11], in younger adults in the US, we hope that our current findings of an increasing incidence of RNET in younger adults will help guide health care screening guidelines and policies toward further investigations on this topic. Future studies are needed to assess the risk factors associated with the revealed trends and to evaluate the RNET mortality and outcomes in demographic-specific populations.

## 5. Conclusions

Our nationwide analysis of the USCS database, covering approximately 98% of the US population, has found that RNET incidence trends have been steady over the past two decades in older adults, while the rates have been increasing in younger adults. The greatest difference between older and younger adults seemed to be arising from younger women and was seen in NHW and NHB patients, as well as in H and NHAPI patients in most years. The increasing trend of RNET in younger adults was mostly driven by tumors diagnosed at an early stage. While this increase can be partially attributed to the increased detection of RNET due to improvements in screening modalities, it can also be a true increase, especially with growing data showing an increase in a variety of gastrointestinal malignancies in younger adults. The exact causes of the revealed trend are unclear. It may be driven by age-specific exposure or response to risk factors that are disproportionally affecting younger adults. The study outlines the imperative need for future studies to investigate the risk factors associated with the increasing incidence of RNET in younger adults, especially in younger women.

## Figures and Tables

**Table 1:** Age-specific trends for rectal neuroendocrine tumors (RNET) incidence rates among men and women.

Age Group, y	Cancer Cases (N = 59,846) ^a^	Trends ^b^			Age-Specific AAPC Difference ^c^ (95% CI)	Pairwise Comparison *p*-Values		
		Time Period	APC (95% CI)	AAPC (95% CI)		Age-Specific AAPC Difference	Coincidence ^d^	Parallelism ^e^
Both Sexes								
Younger Adults	28,963 (48.4%)	2001–2008	8.48 (7.56 to 9.41)	3.12 (2.14 to 4.11)	4.22 (2.33 to 6.10)	<0.001	<0.001	<0.001
		2008–2011	−1.44 (−6.55 to 3.96)					
		2011–2018	4.19 (3.30 to 5.08)					
		2018–2020	−10.85 (−15.41 to −6.05)					
Older Adults	30,869 (51.6%)	2001–2018	0.64 (0.05 to 1.23)	−1.10 (−2.69 to 0.52)				
		2018–2020	−14.70 (−27.34 to 0.13)					
Men								
Younger Men	13,898 (23.2%)	2001–2007	8.03 (5.43 to 10.70)	3.08 (1.67 to 4.51)	3.10 (0.65 to 5.55)	0.01	<0.001	<0.001
		2007–2018	2.94 (2.04 to 3.84)					
		2018–2020	−9.79 (−19.66 to 1.30)					
Older Men	15,865 (26.5%)	2001–2003	14.76 (−2.56 to 35.15)	−0.02 (−2.00 to 2.00)				
		2003–2018	0.28 (−0.32 to 0.87)					
		2018–2020	−14.81 (−24.87 to −3.41)					
Women								
Younger Women	15,065 (25.2%)	2001–2008	8.58 (6.29 to 10.93)	3.31 (0.98 to 5.70)	4.41 (1.53 to 7.30)	0.003	<0.001	<0.001
		2008–2011	−1.93 (−14.16 to 12.05)					
		2011–2017	4.78 (1.82 to 7.84)					
		2017–2020	−5.80 (−11.78 to 0.59)					
Older Women	15,004 (25.1%)	2001–2018	0.30 (−0.26 to 0.87)	−1.10 (−2.75 to 0.57)				

^a^ The data are presented as count numbers followed by their respective percentages relative to the total cases of RNET cancer in the database. ^b^ The time-trends were analyzed using the Joinpoint Regression Program (v4.9.0.1, NCI), with a maximum of three joinpoints allowed (four-line segments). ^c^ A positive value demonstrates a higher AAPC in younger adults compared to older adults. ^d^ Tests whether the age-specific trends were identical. A significant *p*-value demonstrates that the trends were not equivalent (i.e., they had varying incidence rates and coincidence was rejected). ^e^ Tests whether the age-specific trends were parallel. A significant *p*-value indicates non-parallel trends (i.e., parallelism was rejected).

**Table 2:** Age-Specific Time-Trends for Rectal Neuroendocrine Tumors (RNET) Incidence Rates Among Different Race Groups and per Stage at Diagnosis. ^a^ Data are presented as count numbers followed by percentages of the count numbers from the total cases of RNET cancer in the database. ^b^ Time-trends were computed using Joinpoint Regression Program (v4.9.0.1, NCI) with 3 maximum joinpoints allowed (4-line segments). ^c^ A positive value indicates a greater AAPC in younger adults compared to older adults. ^d^ Tests whether age-specific trends were identical. A significant P-value indicates that the trends were not identical (i.e., they had different incidence rates and coincidence was rejected). ^e^ Tests whether age-specific trends were parallel. A significant P-value indicates that the trends were not parallel (i.e., parallelism was rejected).

Age Group, y	Cancer Cases (N = 53,188) ^a^	Trends ^b^			Age-Specific AAPC Difference ^c^ (95% CI)	Pairwise Comparison *p*-Values		
		Time Period	APC (95% CI)	AAPC (95% CI)		Age-Specific AAPC Difference	Coincidence ^d^	Parallelism ^e^
Race								
Non-Hispanic White								
Younger Adults	13,827 (23.1%)	2001–2007	7.12 (4.81 to 9.47)	2.83 (1.47 to 4.21)	4.89 (2.47 to 7.32)	<0.001	<0.001	<0.001
		2007–2018	2.29 (1.42 to 3.16)					
		2018–2020	−6.33 (−16.55 to 5.13)					
Older Adults	15,886 (26.5%)	2001–2018	−0.55 (−1.22 to 0.14)	−2.06 (−4.05 to −0.04)				
		2018–2020	−14.06 (−29.85 to 5.30)					
Non-Hispanic Black								
Younger Adults	7963 (12.9%)	2001–2009	8.43 (6.39 to 10.49)	2.90 (0.28 to 5.58)	3.33 (0.25 to 6.40)	0.03	<0.001	<0.001
		2009–2012	−2.97 (−15.54 to 11.48)					
		2012–2017	5.63 (1.39 to 10.04)					
		2017–2020	−8.31 (−19.80 to 4.84)					
Older Adults	8330 (14.0%)	2001–2007	3.69 (0.33 to 7.17)	−0.43 (−1.99 to 1.15)				
		2007–2017	−0.09 (−1.48 to 1.33)					
		2017–2020	−9.25 (−15.91 to −2.06)					
Hispanic								
Younger Adults	3640 (6.1%)	2001–2018	3.77 (2.67 to 4.90)	2.03 (−0.44 to 4.56)	3.62 (−0.08 to 7.31)	0.05	<0.001	<0.001
		2018–2020	−11.68 (−30.25 to 11.83)					
Older Adults	3160 (5.3%)	2001–2018	0.95 (−0.17 to 2.08)	−1.59 (−4.28 to 1.18)				
		2018–2020	−20.73 (−39.48 to 3.83)					
Non-Hispanic Asian/Pacific Islander								
Younger Adults	2287 (3.8%)	2001–2018	3.16 (1.87 to 4.48)	0.81 (−2.28 to 4.00)	3.49 (1.22 to 5.76)	0.36	<0.001	0.15
		2018–2020	−17.11 (−38.80 to 12.26)					
Older Adults	2289 (3.8%)	2001–2016	1.60 (−0.36 to 3.59)	−1.14 (−3.90 to 1.70)				
		2016–2020	−10.75 (−21.37 to 1.32)					
Stage at Diagnosis								
Early Stage								
Younger Adults	22,371 (37.4%)	2001–2007	9.68 (7.48 to 11.93)	3.47 (2.31 to 4.64)	3.93 (1.82 to 6.05)	<0.001	<0.001	<0.001
		2007–2018	2.54 (1.81 to 3.27)					
		2018–2020	−8.70 (−16.93 to 0.36)					
Older Adults	22,763 (38.0%)	2001–2007	4.46 (1.36 to 7.66)	−0.46 (−2.21 to 1.32)				
		2007–2018	−0.41 (−1.52 to 0.71)					
		2018–2020	−14.16 (−26.26 to −0.07)					
Late Stage								
Younger Adults	1300 (2.2%)	2001–2020	3.43 (1.96 to 4.92)	3.43 (1.96 to 4.92)	2.44 (0.79 to 4.10)	0.004	<0.001	0.001
Older Adults	2712 (4.5%)	2001–2020	0.99 (0.02 to 1.97)	0.99 (0.02 to 1.97)				

**Table 3:** Age-specific time-trends for microscopically confirmed rectal neuroendocrine tumors (RNET) incidence rates among men and women. ^a^ Count numbers followed by percentages from the total cases of RNET cancer. ^b^ The Joinpoint Regression Program (v4.9.0.1, NCI) was used to estimate time-trends with three maximum joinpoints allowed (four-line segments). ^c^ A positive value suggests a greater AAPC in younger adults. ^d^ Evaluates whether the age-specific trends were identical. ^e^ Evaluates whether the age-specific trends were parallel.

Age Group, y	Cancer Cases (N = 59,846) ^a^	Trends ^b^			Age-Specific AAPC Difference ^c^ (95% CI)	Pairwise Comparison *p*-Values		
		Time Period	APC (95% CI)	AAPC (95% CI)		Age-Specific AAPC Difference	Coincidence ^d^	Parallelism ^e^
Both Sexes								
Younger Adults	28,890 (48.3%)	2001–2008	8.48 (7.53 to 9.43)	3.10 (2.10 to 4.12)	4.20 (2.29 to 6.10)	<0.001	<0.001	<0.001
		2008–2011	−1.44 (−6.70 to 4.12)					
		2011–2018	4.20 (3.29 to 5.12)					
		2018–2020	−11.02 (−15.71 to −6.07)					
Older Adults	30,772 (51.4%)	2001–2018	0.63 (0.04 to 1.23)	−1.10 (−2.70 to 0.53)				
		2018–2020	−14.62 (−27.31 to 0.28)					

## Data Availability

The data used in this study are publicly available and can be obtained from the United States Cancer Statistics’ website.
